# Integrated Phytochemical Analysis Based on UPLC-Q-TOF-MS/MS, Network Pharmacology, and Experiment Verification to Explore the Potential Mechanism of *Platycodon grandiflorum* for Chronic Bronchitis

**DOI:** 10.3389/fphar.2020.564131

**Published:** 2020-09-08

**Authors:** Yaling Deng, Hongmin Ren, Xianwen Ye, Lanting Xia, Minmin Liu, Ying Liu, Ming Yang, Songhong Yang, Xide Ye, Jinlian Zhang

**Affiliations:** ^1^ Pharmacy College, Jiangxi University of Traditional Chinese Medicine, Nanchang, China; ^2^ Key Laboratory of Modern Preparation of Traditional Chinese Medicine, Ministry of Education, Jiangxi University of Traditional Chinese Medicine, Nanchang, China

**Keywords:** chemical ingredient, chronic bronchitis, experiment verification, mechanism of action, network pharmacology, *Platycodon grandiflorum*

## Abstract

**Background and Aim:**

*Platycodon grandiflorum* (PG) has been widely used for treating chronic bronchitis (CB). However, the material basis and underlying mechanism of action of PG against CB have not yet been elucidated.

**Methods:**

To analyze the ingredients in PG, ultraperformance liquid chromatography-quadrupole-time-of-flight tandem mass (UPLC-Q-TOF-MS/MS) technology was performed. Subsequently, using data mining and network pharmacology methodology, combined with Discovery Studio 2016 (DS), Cytoscape v3.7.1, and other software, active ingredients, drug-disease targets, and key pathways of PG in the treatment of CB were evaluated. Finally, the reliability of the core targets was evaluated using molecular docking technology and *in vitro* studies.

**Results:**

A total of 36 compounds were identified in PG. According to the basic properties of the compounds, 10 major active ingredients, including platycodin D, were obtained. Based on the data mining approach, the Traditional Chinese Medicine Systems Pharmacology Database, and the Analysis Platform (TCMSP), GeneCards, and other databases were used to obtain targets related to the active ingredients of PG and CB. Network analysis was performed on 144 overlapping gene symbols, and twenty core targets, including interleukin-6 (IL-6) and tumor necrosis factor (TNF), which indicated that the potential signaling pathway that was most relevant to the treatment of CB was the IL-17 signaling pathway.

**Conclusion:**

In this study, ingredient analysis, network pharmacology analysis, and experiment verification were combined, and revealed that PG can be used to treat CB by reducing inflammation. Our findings provide novel insight into the mechanism of action of Chinese medicine. Furthermore, our data are of value for the research and development of novel drugs and the application thereof.

## Introduction

Chronic bronchitis (CB) is chronic, nonspecific inflammation of the trachea, bronchial mucosa, and surrounding tissues, which may be due to infection or noninfection (allergies, oxidative stress) (Malesker et al., 2020). CB is one of the common clinical diseases and has a high incidence in the middle-aged and elderly population. According to statistics, the prevalence of CB in China is about 4.0%, among which 10% to 15% is accounted for by elderly patients, and the incidence is increasing (Luo et al., 2019). The main clinical manifestations of patients are cough, phlegm, or wheezing (Zhang, 2019). The pathological changes mainly involve damage of the epithelium of the central airways. Infiltration of inflammatory cells and hypertrophy of smooth muscle cells further leads to increased mucus secretion, decreased immune function of the epithelium, and ultimately leads to airway remodeling (You, 2014). In Chinese medicine, CB can be divided into six types: phlegm dampness lung type, external cold cohesion type, phlegm heat closed lung type, lung and spleen qi deficiency type, lung and kidney weakness type, and spleen and kidney yang deficiency type. Only when symptomatic treatment is performed, the recurrence of CB can be relieved from the root cause (Weng et al., 2014; Zhu, 2017; Bai and Li, 2019). At present, Western medicine and Chinese medicine have been shown to be successful in the treatment of CB (Sun et al., 2015; Zhu, 2017). Treatment involving Western medicine mainly uses antiinfective, antiallergic, relieves bronchial smooth muscle spasm and other antitussive, phlegm-reducing drugs to treat CB, including ambroxol hydrochloride (Sun et al., 2015), budesonide (Wesseling et al., 1991), and levofloxacin (Blasi et al., 2013). Short-term use of Western medicine may temporarily relieve symptoms, but due to the long course of the disease, long-term use has several shortcomings, including toxic side effects, patient intolerance, and high costs. In addition, patients with CB are often frail and other systems are affected, therefore, they are often forced to discontinue treatment because of the toxic side effects of certain drugs (You, 2014; Jin, 2019). In view of the shortcomings of Western medicine, it is of utmost importance to develop drugs that can safely and effectively treat CB. For the treatment of CB, traditional Chinese medicine (TCM) is mainly used to clear the lungs and phlegm, spleen and kidney function, and has effectively relieved symptoms, including cough and phlegm. Long-term use of TCM can effectively enhance human immunity and reduce the frequency of attacks of CB, including Shegan Mahuang Decoction (Bai and Li, 2019), Zhi Chou San (Bai and Li, 2019), and Tianxing Kechuan Patches (Fan et al., 2012). Compared with Western medicine, TCM has the characteristics of a multiingredient and multitarget action. In addition, it can perform overall regulation and multitarget intervention on CB (Zhao and Li, 2018).

*Platycodon grandiflorum* (PG) is the dried root of campanulaceae, and PG extracted from platycodon has been shown to have a good effect on ventilating lung and eliminating phlegm. According to the TCM theory, PG mainly acts on the lung and its related structures. It has a cough-relieving effect and has been used to treat CB with good curative effect (Chen et al., 2010; Chen et al., 2013; He et al., 2013). PG contains triterpenoid saponins, flavonoids, phenolic acids, polyacetylene, and sterols (Deng et al., 2020; Ji et al., 2020). Among them, platycodin D is one of the main active ingredients (Deng et al., 2020). Multiingredients are both independent and have connections, and may lead to cross-links between targets. Therefore, for the treatment of CB, PG should act as a multiingredient and multitarget in synergy to exert drug effects, which means that its mechanism of action is complex. Thus, a method that establishes the relationship between ingredients, targets, and diseases is warranted for exploring the underlying mechanism of action by which PG treats CB. Network pharmacology is a theory based on systems biology, and emphasizes the multichannel regulation of signaling pathways, which coincides with the characteristics of multiingredient-multitargets of TCM (Zhang et al., 2019; Li W. J. et al., 2020; Ou et al., 2020). Network pharmacology integrates TCM, active ingredients of TCM, TCM targets, disease targets, constructs drug-ingredients-gene symbols-disease four-dimensional graphs, and comprehensively analyzes common targets of Chinese medicine ingredients on diseases, and thoroughly analyzes target genes, proteins, and signal pathways, to identify possible mechanism of Chinese medicine treatment to treat diseases (Park et al., 2018; Yang et al., 2019; Ye et al., 2020). Therefore, the network pharmacology approach is a tool that is sufficient to identify the mechanism underlying the treatment of CB by PG.

**Figure 1:**
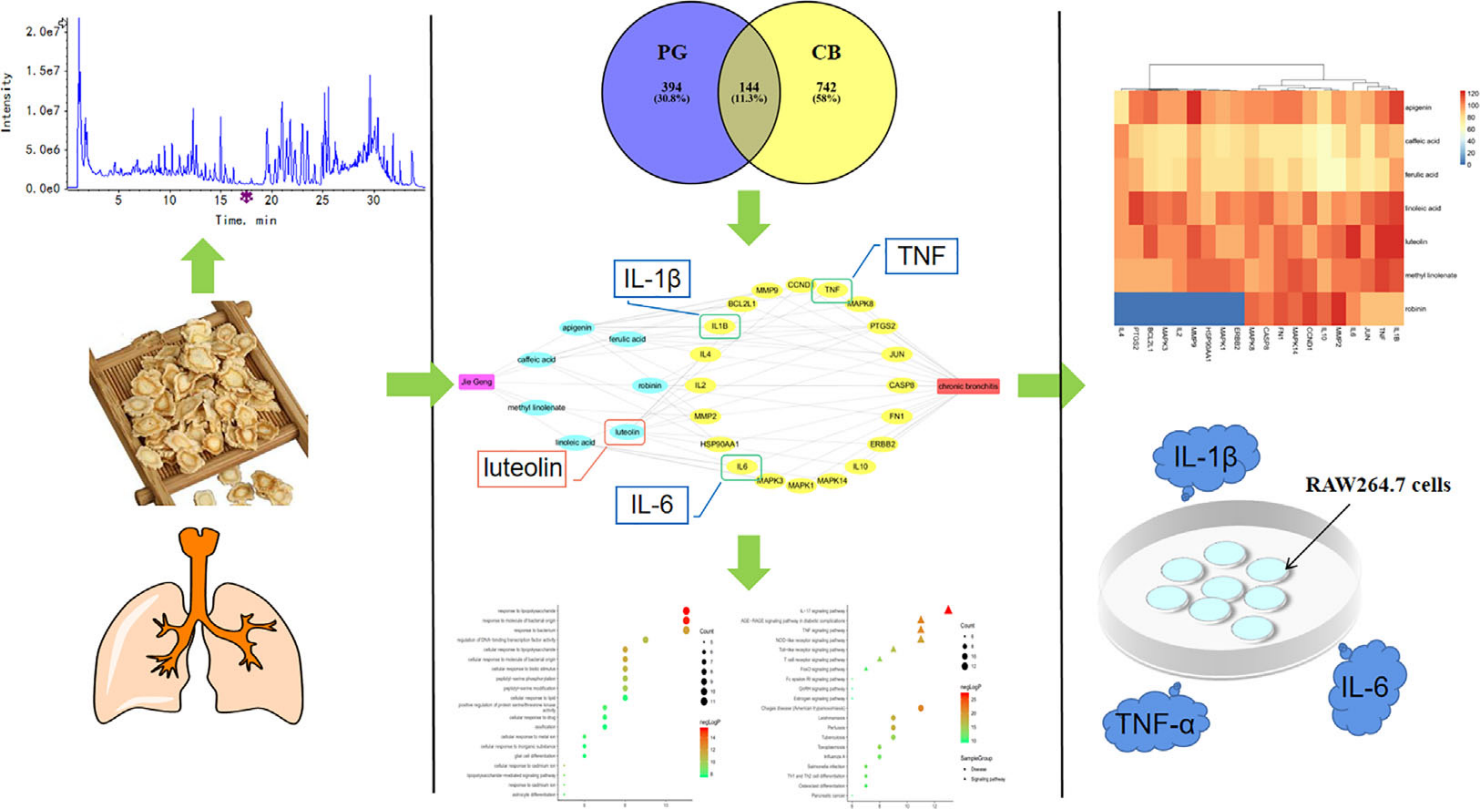
A comprehensive strategy diagram the chemical ingredients analysis, targets prediction, network calculation and experimental validations for investigation the mechanism of action of *Platycodon grandiflorum* (PG) on chronic bronchitis (CB).

PG is mainly produced in the provinces of Northeast, North China, East China, Central China, and Guangdong. There are differences in PG ingredients from different areas. Shandong is one of the genuine producing areas of PG, and the PG produced there has long roots, few bifurcations, and a high content of active ingredients (Zhu et al., 2013). However, the ingredients of PG from Shandong have not yet been systematically analyzed and identified. Therefore, in this study, ingredient analysis of PG produced in Shandong was conducted, and based on the relevant principles and methods of network pharmacology, drug-ingredients-gene symbols-disease (D-I-G-D) network was constructed to explore the potential molecular mechanism for treating CB. Subsequently, the reliability of the core targets was verified by molecular docking verification and *in vitro* studies (**Figure 1**). Our findings will provide a theoretical basis for the clinical application of PG and the development of novel drugs.

## Materials and Methods

### Chemicals and Herb Materials

Methanol, acetonitrile, and formic acid for high performance liquid chromatography (HPLC) were purchased from ACS (Washington D.C., MD, USA). Methanol for herb extraction was purchased from Xilong Scientific Co., Ltd. (Guangdong, China). Ultrapure water was obtained from a Milli-QB system (Bedford, MA, USA). PG pieces were purchased from Jiangxi Jiangzhong Herbal Pieces Co., Ltd. (Jiangxi, China; batch number: 181024).

The original PG medicinal material was purchased from Yiyuan, Shandong province, and was identified as the dried root of PG (Jacq.) A. DC. Campanulaceae by Professor Fu Xiaomei. PG decoction pieces were processed by Jiangxi Jiangzhong TCM Decoction Co., Ltd. according to the processing method of the Chinese Pharmacopeia 2015 edition. Next, dried PG pieces were crushed into powder (40 mesh) and stored in the laboratory of the Jiangxi University of TCM.

As reference standards, 13 pure compounds were used (purity≥98%). Among these compounds, chlorogenic acid (2), caffeic acid (3), ferulic acid (7), lobetyolin (8), luteolin (15), kaempferol (16), apigenin (17), deapio-platycodin D (19), and platycodin D (21) were purchased from Chengdu Chroma-Biotechnology Co., Ltd. (Sichuan, China). Lobetyol (4), rutin (5), 3-O-β-D-glucopyranosyl platycodigenin (30), and linoleic acid (36) were purchased from Sichuan Vicky Biotechnology Co., Ltd. (Sichuan, China).

Enzyme-linked immunosorbent assay (ELISA) kits for tumor necrosis factor-α (TNF-α), interleukin-6 (IL-6), and interleukin-1β (IL-1β) were purchased from Huamei Biotechnology (Wuhan, China). The minimum detectable dose of mouse TNF-α, IL6, and IL-1β is typically less than 3.9 pg/ml, 0.39 pg/ml, and 7.8 pg/ml, respectively. Intra-assay and inter-assay precision of these three ELISA kits is less than 8% and 10%.

### Ultra-Performance Liquid Chromatography-Quadrupole-Time-of-Flight Tandem Mass Analysis

#### Preparation of Standard and Sample Solutions

Ten milligrams of each reference compound (luteolin; chlorogenic acid; caffeic acid; ferulic acid; lobetyolin; kaempferol; apigenin; lobetyol; deapio-platycodin D; linoleic acid; 3-O-β-D-glucopyranosyl platycodigenin; rutin; platycodin D) was weighed, transferred to 10-ml volumetric flasks, methanol was added to reach the volumetric mark, shaken well, and used as a stock solution. Then, the appropriate amount of stock solution was added to a 5-ml volumetric flask, and methanol was added to reach the volumetric mark. The solutions were filtered through 0.22-μm microporous membranes to obtain the standard solutions.

PG powder (2.0 g) was accurately weighed and placed in a round bottom flask with 50 ml 50% methanol, mixed well, soaked for 0.5 h at room temperature, and treated ultrasonically for 30 min using an ultrasonic cleaning instrument (Jiangsu, China). The extract solution was centrifuged at 14,000 rpm for 15 min at room temperature, then filtered through a 0.22-μm microporous membrane before qualitative analysis.

#### Ultra-Performance Liquid Chromatography-Quadrupole-Time-of-Flight Tandem Mass Conditions

Chemical analysis was conducted on a connected system of UPLC (Nexera X2 LC-30A, Shimadzu Corp., Japan)-hybrid triple quadruple time-of-flight mass spectrometer (Triple TOF™ 5600^+^, AB Sciex, Forster City, CA, USA) with an electrospray ionization source (ESI). Acquity UPLC BEH C_18_ column (2.1×100 mm×1.7µm) was used to perform chromatographic separation with a flow rate of 0.25 ml/min at 40°C. A linear gradient program with a mobile phase system including solvent A (100% acetonitrile, v/v) and solvent B (0.01% formic acid in water, v/v) was described in detail: solvent A (5%~23%) for 10 min, (23%~25%) for 6 min, (25%) for 4 min, (25%~29%) for 3 min, (29%~95%) for 7 min, (95%~5%) for 2.1 min, isocratic eluted at 5% for 2.9 min.

The instrumental settings of Q-TOF-MS/MS were as follows: ion source gas 1 (GSI) and gas 2 (GS2) were both set to 50 psi, curtain gas (CUR) was set to 40 psi, ion spray voltage floating (ISVF) was set to 5500 V in the positive mode while 4500 V was set in the negative mode, ion source temperature (TEM) was 500°C, collision energy (CE) was 60 V, collision energy spread (CES) was 15 V, declustering potential (DP) was 100 V, and nitrogen was used as a nebulizer and auxiliary gas. Samples were analyzed in both positive and negative ionization modes with a scanning mas-to-charge (m/z) range from 100 to 1,250. Data were collected in information-dependent acquisition (IDA) mode and analyzed by PeakView^®^1.2 software (AB Sciex, Foster City, CA, USA).

#### Ingredients Identification Analysis

The chemical ingredients of PG were collected from existing databases, including SciFinder (https://scifinder.cas.org/), the Traditional Chinese Medicine Systems Pharmacology Database, and the Analysis Platform (TCMSP, http://lsp.nwu.edu.cn/tcmsp.php) database. Then, a PG ingredients database was established, which contained basic information, such as ingredient name and molecular formula. A total of 161 known ingredients in PG were collected, and specific information is presented in **Schedule 1**. MS data was imported into PeakView^®^ 1.2 for ingredient analysis. Chemical identifications were based on reference standards, chromatographic elution behaviors, chemical ingredient, mass fragment patterns, and mass spectral library (Natural Products HR-MS/MS Spectral Library, Version 1.0, AB Sciex, Forster City, CA, USA).

### Collecting Related Targets for Active Ingredients in PG

In this study, four databases were searched to identify the target of active ingredients in PG. Databases searched included Swiss Target Prediction (http://www.swisstargetprediction.ch/) (Gfeller et al., 2014), Pubchem (https://pubchem.ncbi.nlm.nih.gov/) (Liu et al., 2012), TCMSP (Ru et al., 2014), and Pharmmapper (http://www.lilab-ecust.cn/pharmmapper/) (Liu et al., 2010). Targets were converted into gene symbols by Uniprot (http://www.uniprot.org/), and gene symbols were combined.

### CB-Associated Targets Collection

In this study, “Chronic bronchitis” was used as a keyword to search for relevant CB targets in DisGenet (http://www.disgenet.org/) (Li B. T. et al., 2019; Su et al., 2019) and GeneCards databases (https://www.genecards.org/) (Zhang et al., 2018; Yang et al., 2019).

### Protein-Protein Interaction Network Construction

To obtain overlapping targets, VENNY 2.1 (http://www.liuxiaoyuyuan.cn/) software was used to cross PG-related targets with CB-related targets. Overlapping targets of Chinese medicine-disease were added into STRING11.0 (https://string-db.org/) (Snel et al., 2000; Yang et al., 2019), and the screening condition used was “Homo sapiens”, the minimum interaction score was 0.4, and the results were saved. The resulting file was imported into Cytoscape v3.7.1 software, and the plugin CentiScape was used to calculate the degree centrality (DC). The core target of the protein-protein interaction (PPI) network was filtered (Li Z. et al., 2020).

### D-I-G-D Network Construction

Related files were established of “drug-core ingredients,” “core ingredients-core targets,” and “disease-core targets,” and files were imported into Cytoscape v3.7.1 to build a “drug-ingredients-gene symbols-disease” network.

### Gene Ontology and Kyoto Encyclopedia of Genes and Genomes (KEGG) Pathway Enrichment Analysis

Metascape (http://www.metascape.org/) is a gene annotation tool that integrates multiple authoritative data sources such as gene ontology (GO), Kyoto Encyclopedia of Genes and Genome (KEGG), UniProt, and DrugBank. It not only completes pathway enrichment and bioprocess annotation, but also performs gene-related protein network analysis and drug analysis, and is committed to providing comprehensive and detailed information on each gene (Zhou et al., 2019). On the premise of retaining the advantages of DAVID, Metascape has perfectly made up for its vacancy, and data are frequently updated, which guarantees the timeliness and credibility of the data.

Gene symbols of the core targets were imported into Metascape, and “Homo sapiens” was selected for enrichment analysis to further explain the role of the core targets in gene function and signaling pathways.

### Computational Validation of Ingredients-Target Interactions

In this study, we aimed to ascertain the interaction between active ingredients and their protein targets, and explore their binding modes. Hence, we selected seven core ingredients and twenty core targets for verification of molecular docking. The PDB format of core ingredients was obtained from the Uniprot database, and the X-ray crystal structures were downloaded from the RCSB database (https://www.rcsb.org/). The molecular docking function of Discovery Studio 2016 (DS) was used for ingredient-target molecular docking in the LibDock module.

### Experimental Verification *In* V*itro*

#### Cell Culture

RAW264.7 cells were obtained from the Beijing Beina Chuanglian Biotechnology Research Institute (Beijing, China). Cells were cultured in Dulbecco’s modified Eagle’s medium (DMEM, Solarbio, Beijing, China), supplemented with 10% fetal bovine serum (FBS, Zhejiang, China). Cells were cultured at 37°C and 5% CO_2_.

#### Cell Viability Assay

RAW264.7 cells in the logarithmic phase were seeded at 1×10^4^ cells/well in 96-well plates. After incubation for 24 h, RAW264.7 cells were exposed to luteolin (0, 15, 20, 40, 45, 60, 80, and 100 μM). After treatment for 24 h, 20 μl of Cell Counting Kit (CCK-8) assay solution (Solarbio, Beijing, China) was added to each well, and cells were incubated for 4 h at 37°C and 5% CO_2_. The absorbance at 450 nm was measured by a microplate reader (FLUOstar Omega, LABTECH, Offenburg, Germany). Cell survival was calculated as: absorbance/absorbance of control ×100%.

#### TNF-α, IL-6, and IL-1β Expression

RAW264.7 cells (5×10^3^ cells/well in 96-well plates) were incubated with lipopolysaccharide (LPS; 1 μg/ml) for 24 h, then treated with luteolin (20, 40, or 60 μM) for 24 h. Supernatants were harvested and levels of IL-6, TNF-α, and IL-1β were determined by ELISA.

#### Real-Time Quantitative Polymerase Chain Reaction

Total RNA was extracted using Trizol reagent (Beijing, China), then treated with RNase-free DNase (Promega, Beijing, China), and reverse transcribed with oligo-DT (Beijing, China) using MMLV (TOYOBO, Shanghai, China) reverse transcriptase according to the instructions of the reverse transcription kit. Reverse transcription reaction conditions were as follows: 30°C for 10 min, 42°C for 60 min, 99°C for 5 min, and 4°C for 5 min. The reaction was carried out in a PCR machine (Applied Biosystems, Foster City, CA, USA). Primers were commissioned by Dingguo Changsheng Biotechnology Co., Ltd (Beijing, China) and primers sequences were as follows:

IL-1β: (Forward primer) 5’-TGCCACCTTTTGACAGTGATG-3’
(Reverse primer) 5’-AAGGTCCACGGGAAAGACAC-3’
IL-6: (Forward primer) 5’-ACAAGTCCGGAGAGGAGACT-3’
(Reverse primer) 5’-TGTGACTCCAGCTTATCTCTTGG-3’
TNF: (Forward primer) 5’-ACCCTCACACTCACAAACCA-3’
(Reverse primer) 5’-ACCCTGAGCCATAATCCCCT-3’
GAPDH: (Forward primer) 5’-GGGGTCCCAGCTTAGGTTC A-3’
(Reverse primer) 5’-TTCCCATTCTCGGCCTTGAC-3’

Real-time quantitative polymerase chain reaction (qRT-PCR) was performed by SYBR™ Green Master Mix (Vazyme, Beijing, China) in an QuantStudio 6 Flex system (Applied Biosystems, Foster City, CA, USA). The PCR cycling profile was as follows: one cycle at 95°C for 3 min and 72°C for 10 min, 35 cycles at 94°C, 60°C, and 72°C for 30 s. Fluorescence signals were detected using the QuantStudio 6 Flex system. Gene-expression data were normalized to that of the endogenous control GAPDH. The 2^-△△Ct^ method was used as the basis for relative gene expression.

### Statistical Analyses

Data were analyzed by SPSS 25.0 (SPSS Inc., Chicago, IL, USA) and expressed as the mean ± standard deviation (SD). All experiments were performed in triplicate. Data were analyzed by one-way analysis of variance (ANOVA) followed by least significant difference (LSD) testing. *p*<0.05 was considered statistically significant.

## Results

### Identification of the Chemical Constituents in PG by Ultraperformance Liquid Chromatography-Quadrupole-Time-of-Flight Tandem Mass

**Figure 2:**
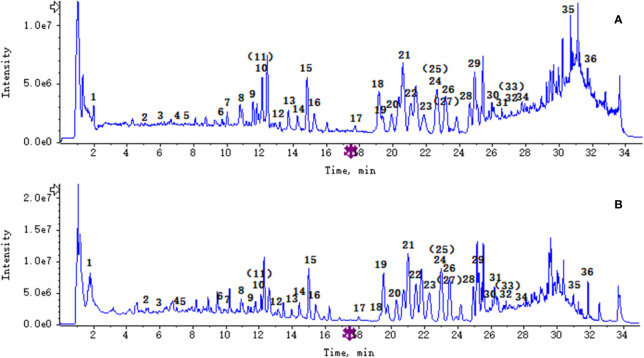
The total ion chromatograms total ion chromatogram (TICs) of *Platycodon grandiflorum* (PG) by ultra-performance liquid chromatography-quadrupole-time-of-flight tandem mass (UPLC/Q-TOF-MS/MS). **(A)** TIC of PG in positive ion mode; **(B)** TIC of PG in negative ion mode.

**Figure 3:**
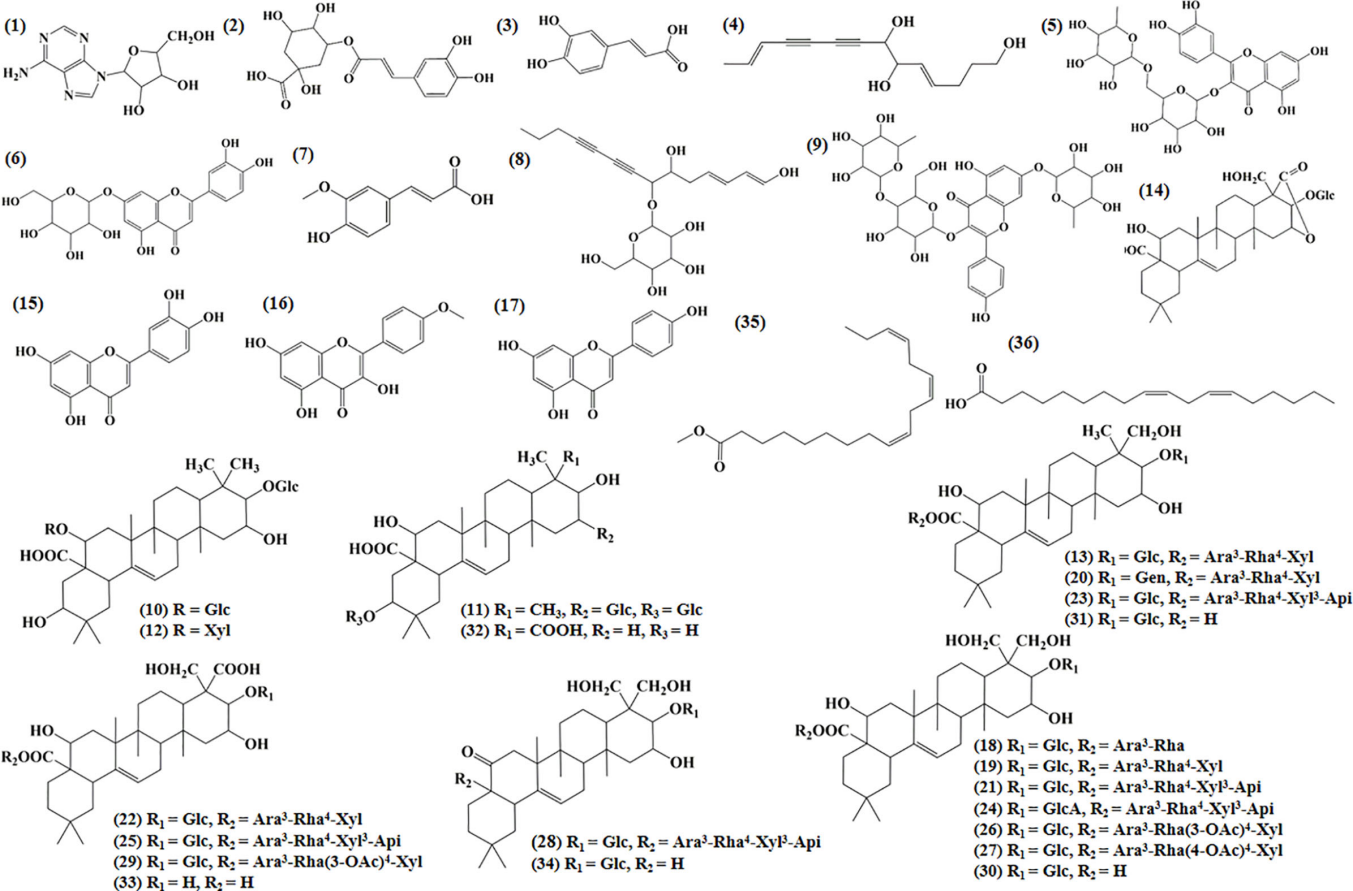
Structural of compounds in *Platycodon grandiflorum* (PG).

Ultraperformance liquid chromatography-quadrupole-time-of-flight tandem mass (UPLC-Q-TOF-MS/MS) is a high-throughput analytical technology that has rapidly developed in the past decade, and is widely used in the fields of environmental science, medicine, drug research, and others (Ren et al., 2020). Typical total ion chromatograms (TICs) of nonvolatile ingredients extracted by PG are presented in **Figure 2**. Although TICs are complex, most of the chromatographic peaks are well separated. A total of 36 chemical constituents were identified in the 50% aqueous methanol extract of PG based on the reference standards, chromatographic elution behaviors, chemical ingredient, mass fragment patterns as well as mass spectral library with the high resolution UPLC-Q-TOF MS/MS system, including triterpene saponins, flavonoids, phenolic acids, and polyacetylene. Among them, 13 compounds (2, 3, 4, 5, 7, 8, 15, 16, 17, 19, 21, 30, and 36) were identified by comparing with the reference standards. By comparing references (Guo, 2007; Wang et al., 2017; Wang, 2018; Deng et al., 2020) and mass fragmentation patterns, another 23 ingredients were identified. Detailed information of the 36 compounds, including chemical name, formula, molecular weight, retention time (t_R_), fragment ions, and structural formula are presented in **Table 1** and **Figure 3**. Due to the limitations of mass spectrometry analysis, several components with high response values have not been effectively identified, see **Schedule 2** for details.

**Table 1:** Components identification results of 50% methanol extracts from *Platycodon grandiflorum* (PG).

NO	t_R_/min	Molecular Formula	ESI-MS	ESI-MS/MS	Error (ppm)	Identity	Ref.
1	1.35	C^10^H^13^N^5O4^	268.10403[M+H]+	136.0621, 119.0354	−0.5	adenosine	(Li Z. et al., 2020)
2	5.1	C^16^H^18O9^	353.08781[M-H]-	191.0577	−3.6	chlorogenic acid	(Li Z. et al., 2020)
3	5.97	C^9^H^8O4^	179.03498[M-H]-	135.0502, 107.0578, 117.0441	3.9	caffeic acid	(Li Z. et al., 2020)
4	7.21	C^14^H^18O3^	235.13287[M+H]+	115.0607	−2.6	lobetyol	(Li Z. et al., 2020)
5	7.32	C^27^H^30^O^16^	611.16066[M+H]+	287.0541	−0.8	rutin	(Li Z. et al., 2020)
6	9.58	C^21^H^20^O^11^	447.09329[M-H]-	285.0386	−3.9	luteolin-7-0-glucoside	(Deng et al., 2020)
7	10	C^10^H^10^O^4^	193.05063[M-H]-	133.0344	0.9	ferulic acid	(Deng et al., 2020)
8	10.72	C^20^H^28^O^8^	397.18569[M+H]+	216.9751, 198.9699, 165.0696, 153.0670, 141.0703, 127.0592, 115.0598, 105.0346	−2	lobetyolin	(Li et al., 2020)
9	11.65	C^33^H^40^O^19^	739.20910[M-H]-	221.0714, 179.0589, 161.0497	−3	robinin	(Zhou et al., 2019)
10	11.99	C^42^H^68^O^16^	827.44346[M-H]-	827.4407	−2	platycodon A	(Deng et al., 2020)
11	11.99	C^42^H^68^O^16^	827.44346[M-H]-	827.4407	−2	platycosaponin A	(Li et al., 2020)
12	13.04	C^41^H^66^O^15^	797.43290[M-H]-	797.4297	−2.1	platycodon B	(Deng et al., 2020)
13	14.09	C^52^H^84^O^23^	1075.53306[M-H]-	1075.5283, 665.3873, 337.1133	−2.7	platycoside J	(Deng et al., 2020)
14	14.55	C^36^H^54^O^12^	677.35425[M-H]-	677.352	−3.3	platycoside M1	(Deng et al., 2020)
15	14.61	C^15^H^10^O^6^	285.04046[M-H]-	133.0303	−0.6	luteolin	(Deng et al., 2020)
16	14.65	C^15^H^10^O^6^	285.04046[M-H]-	133.0308	−0.3	kaempferol	(Deng et al., 2020)
17	18.55	C^15^H^10^O^5^	269.04555[M-H]-	117.0384, 107.0149	−0.8	apigenin	(Deng et al., 2020)
18	19.07	C^47^H^76^O^20^	959.48572[M-H]-	681.3815	−2.7	platycoside F	(Li Z. et al., 2020)
19	19.51	C^52^H^84^O^24^	1091.52798[M-H]-	1091.5313, 681.3853, 337.1140	−2.5	deapio-platycodin D	(Li Z. et al., 2020)
20	19.99	C^58^H^94^O^28^	1237.58589[M-H]-	1237.5459	−2.1	platycoside H	(Deng et al., 2020)
21	20.73	C^57^H^92^O^28^	1223.57024[M-H]-	1223.5671, 681.3855, 469.1559	−3.7	platycodin D	(Li Z. et al., 2020)
22	21.27	C^52^H^82^O^25^	1105.50724[M-H]-	1105.5025, 1075.4941, 895.4301, 485.2880	−2.8	platyconic acid C	(Li Z. et al., 2020)
23	21.95	C^57^H^92^O^27^	1207.57532[M-H]-	1207.5717, 665.3907, 541.1756, 469.1544	−3.2	polygalacin D	(Deng et al., 2020)
24	22.74	C^57^H^90^O^29^	1237.5495[M-H]-	1237.5440, 1207.5346, 1027.4720	−2.2	platycodin J	(Deng et al., 2020)
25	22.74	C^57^H^90^O^29^	1237.5495[M-H]-	1237.5440, 1207.5346, 1027.4720	−2.2	platyconic acid A	(Li Z. et al., 2020)
26	23.73	C^54^H^86^O^25^	1133.53854[M-H]-	1133.5343, 1091.5274, 723.3920, 691.3822, 663.3695, 501.3229, 337.1144	−2.4	platycoside B	(Li Z. et al., 2020)
27	23.73	C^54^H^86^O^25^	1133.53854[M-H]-	1133.5343, 1091.5274, 723.3920, 691.3822, 663.3695, 501.3229, 337.1144	−2.4	platycoside C	(Deng et al., 2020)
28	24.79	C^57^H^90^O^28^	1221.55459[M-H]-	1221.5481, 469.1542	−2.5	16-OXO-platycodin D	(Deng et al., 2020)
29	25.11	C^54^H^84^O^26^	1147.51781[M-H]-	1147.5104, 1117.5032, 937.1142, 485.2896	−2.2	platyconic acid D	(Li Z. et al., 2020)
30	25.84	C^36^H^58^O^12^	681.38555[M-H]-	681.3817, 635.3761, 471.3072, 457.3307, 379.2971	−4.3	3-O-β-D-glucopyranosyl platycodigenin	(Deng et al., 2020)
31	26.14	C^36^H^58^O^11^	665.39064[M-H]-	665.3867, 619.3991, 503.3352, 441.3325, 101.0291	−3.4	3-O-β-D-glucopyranosyl polygalacic acid	(Deng et al., 2020)
32	26.92	C^30^H^46^O^8^	533.31199[M-H]-	533.3092, 469.2915	−3.6	platycogenic acid B	(Deng et al., 2020)
33	26.92	C^30^H^46^O^8^	533.31199[M-H]-	533.3092, 485.2892, 469.2915, 441.3001, 377.2838	−3.6	platycogenic acid A	(Deng et al., 2020)
34	27.81	C^35^H^56^O^10^	635.38007[M-H]-	473.3247, 443.3125, 425.3030, 379.2631, 217.1586	−4	platycodonoids B	(Deng et al., 2020)
35	30.76	C^19^H^32^O^2^	293.24751[M+H]+	145.1014, 131.0870, 119.0871, 105.0720	−3.8	methyl linolenate	(Ren et al., 2020)
36	31.87	C^18^H^32^O^2^	279.23295[M-H]-	279.2246	0.8	linoleic acid	(Zhou et al., 2019)

### Screening Active Ingredients in PG

In general, molecules with “Oral bioavailability”(OB) ≥ 30% and “Drug likeness”(DL) ≥ 0.18 are considered to have better pharmacological effects, therefore this standard is also often used when screening for the active ingredients of drugs (Alam et al., 2015; Li Z. et al., 2020; Meng et al., 2020; Ye et al., 2020).

To identify the active constituents of PG, DS was used to predict the Absorption-Distribution-Metabolism-Excretion-Toxicity (ADMET) properties of chemical constituents, and apigenin, caffeic acid, kaempferol, lobetyol, linoleic acid, methyl linolenate, and ferulic acid were screened. To more comprehensively screen the active ingredients of PG, some ingredients did not meet the DS screening criteria, however, they remained active ingredients. For example, although luteolin and robinin did not meet the DS screening criteria, luteolin (OB=36.16%, DL=0.25) and robinin (OB=39.84%, DL=0.71) were obtained from TCMSP, and for both, the OB was greater than 30%, and the compound DL was greater than 0.18, thus they were retained as active ingredients. In previous studies, it was shown that PG has a preventive effect on CB (Chen et al., 2010; Chen et al., 2013; He et al., 2013), and platycodin D is one of the main active ingredients in PG. In summary, in this study, 10 ingredients were selected as active ingredients of PG (**Table 2**).

**Table 2:** Information on the 10 main active ingredients in *Platycodon grandiflorum* (PG).

**NO**	**CAS**	**Compounds**	**Molecular Formula**	**Structural formula**
1	58479-68-8	platycodin D	C_57_H_92_O_28_	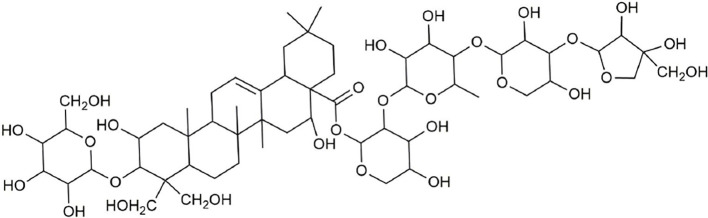
2	1135-24-6	ferulic acid	C_10_H_10_O_4_	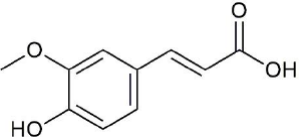
3	520-36-5	apigenin	C_15_H_10_O_5_	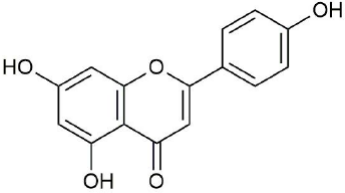
4	520-18-3	kaempferol	C_15_H_10_O_6_	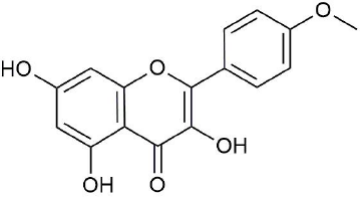
5	331-39-5	caffeic acid	C_9_H_8_O_4_	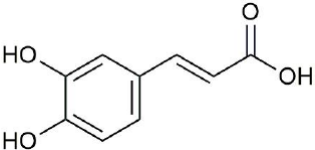
6	7361-80-0	methyl linolenate	C_19_H_32_O_2_	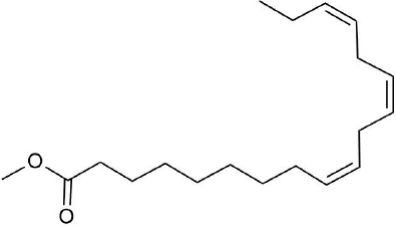
7	60-33-3	linoleic acid	C_18_H_32_O_2_	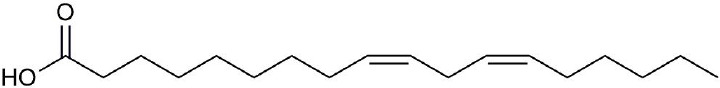
8	136171-87-4	lobetyol	C_14_H_18_O_3_	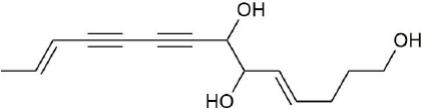
9	491-70-3	luteolin	C_15_H_10_O_6_	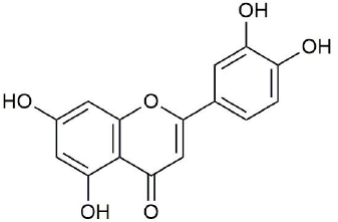
10	301-19-9	robinin	C_33_H_40_O_19_	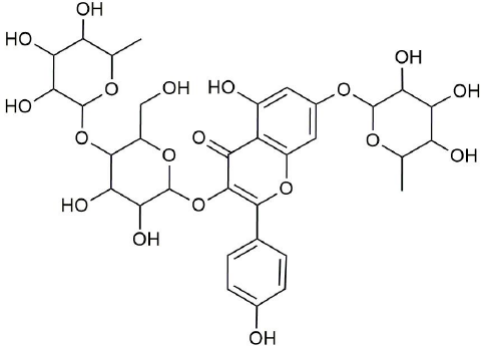

### PPI Network Analysis

The number of drug-targets obtained from Swiss Target Prediction, Pubchem, TCMSP, and Pharmmapper were 58, 193, 274, and 190, respectively. Using “Chronic bronchitis” as a keyword, 876 and 65 disease-targets were obtained from GeneCards and DisGenet databases, respectively. Moreover, after removing duplicate targets, a total of 538 drug-targets and 886 disease-targets were obtained (**Schedule 3** and **Schedule 4**).

**Figure 4:**
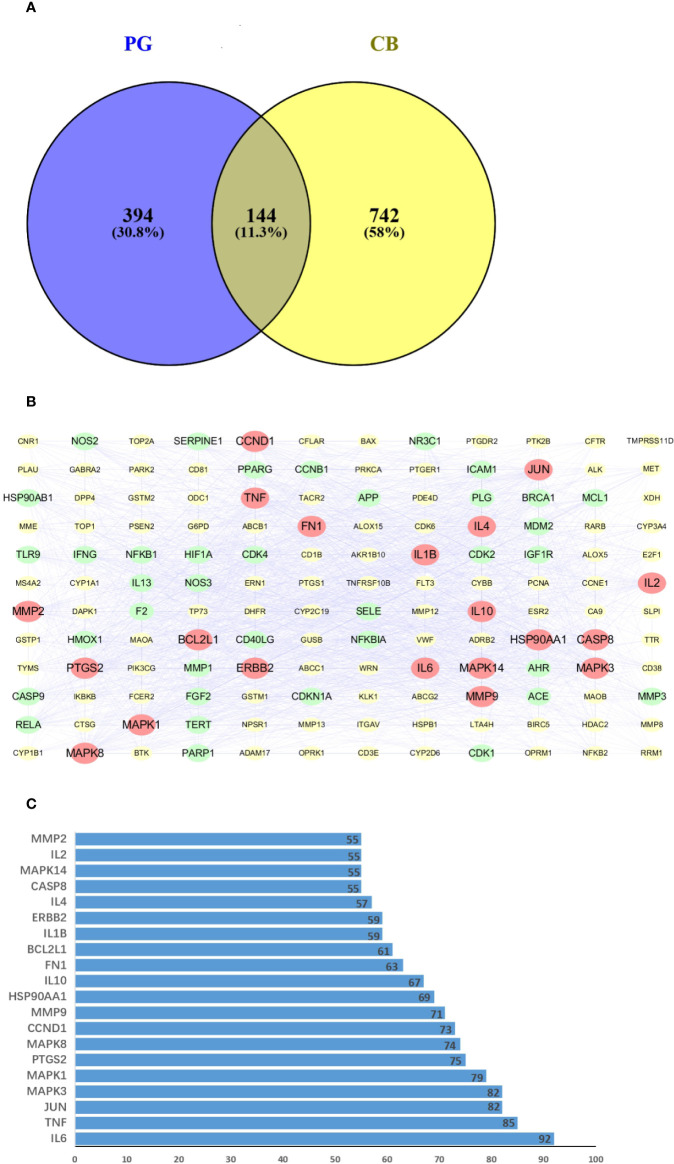
**(A)** Venn diagram of related targets of *Platycodon grandiflorum* (PG) and chronic bronchitis (CB). **(B)** Protein-protein interaction (PPI) network of overlapping targets between drug and disease, the yellow boxes represent the nonpotential target, the green boxes represent the potential target, and the red boxes represent the core target. **(C)** Number of adjacent nodes of overlapping targets between drug and disease, the X-axis indicates the number of neighboring proteins of the target, the Y-axis indicates different targets.

Using VENNY 2.1 software to cross drug-related targets with disease-related targets, and to create the drug-disease overlapping targets Venny diagram (**Figure 4A**), 144 overlapping targets were obtained (**Schedule 5**).

To construct a PPI network consisting of 144 nodes and 2087 edges, a total of 144 drug-disease overlapping targets were introduced into STRING. PPI network diagrams were imported into Cytoscape software for visualization (**Figure 4B**). The data showed that TMPRSS11D did not interact with other targets. CentiScape calculated the average DC value of the intersection target at 29.189, and targets with a DC value greater than 57 were considered potential targets. The top 20 potential targets of DC values were selected as the core target (**Figure 4C**), and IL-6 and TNF were related to more than 85 proteins. These targets will be studied with a focus on protein interaction.

### D-I-G-D Network Analysis

**Figure 5:**
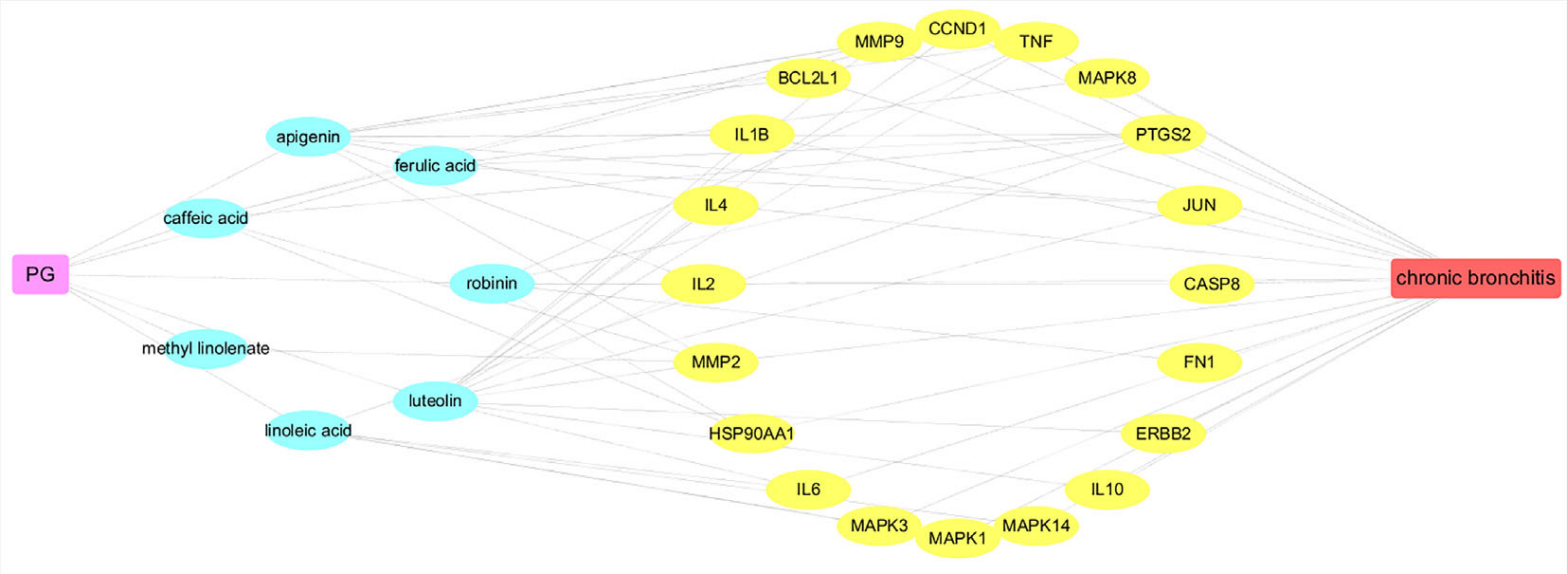
The Drug-Ingredients-Gene symbols-Disease (D-I-G-D) Network.

To elucidate the underlying mechanism of PG on the treatment of CB, Cytoscape v3.7.1 software was employed to construct a drug-ingredients-gene symbols-disease network, as presented in **Figure 5**, in which the fuchsia node represented PG, the seven blue nodes represented seven core ingredients of PG, the 20 yellow nodes represented 20 core gene symbols between PG and CB, the red node represented CB, and edges represented targets interacting with each other.

Network analysis was conducted through evaluating centralization and heterogeneity (0.587 and 0.821) and it was found that some nodes were more concentrated in the network than others. The network contained ingredients with multiple targets, such as luteolin (degree=12), apigenin (degree=11), and robinin (degree=7). This indicated that PG can act on multiple targets with the same active ingredient. For example, if used to treat CB, luteolin, and apigenin may have an effect on IL-6, TNF, and IL-1β. In a previous study, Thaise and colleagues showed that luteolin can reduce inflammatory responses by inhibiting the expression of IL-6, IL-1β, and TNF-α (Boeing et al., 2020). In addition, Hwa and colleagues found that luteolin can effectively inhibit the activity of inflammatory factors, such as IL-1β and TNF-α (Jeon et al., 2014). Likewise, in many reports, it has been described that apigenin can effectively inhibit inflammation. Apigenin could further downregulate the NF-κB signal, inhibit the expression of IL-6, IL-1β, and TNF-α, so as to achieve antiinflammatory effects (Dang et al., 2018; Park et al., 2020).

### GO and KEGG Pathway Enrichment Analysis

**Figure 6:**
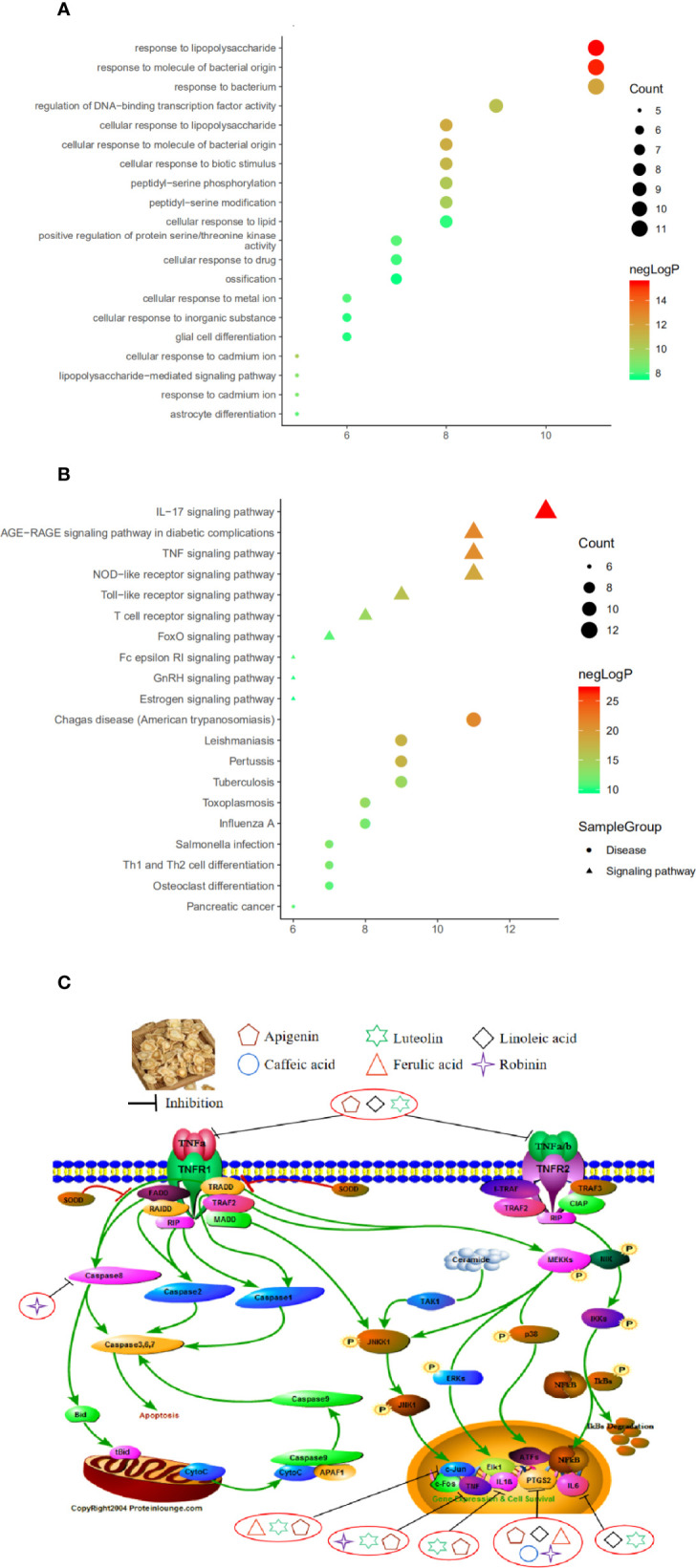
**(A)** Bubble chart of gene ontology (GO) function enrichment of core targets. **(B)** Bubble chart of Kyoto Encyclopedia of Genes and Genomes (KEGG) enrichment of core targets. **(C)** The potential mechanism of *Platycodon grandiflorum* (PG) treatment of chronic bronchitis (CB).

Used Metascape to perform pathway and process enrichment analysis on 20 core targets, screening results with a p-value<0.01, a minimum count of 3, and an enrichment factor>1.5, resulted in 710 GO biological process and 113 KEGG pathway enrichment results. The results showed that GO biological processes were related to the treatment of CB, and included response to: LPS (GO:0032496), response to a molecule of bacterial origin (GO:0002237), regulation of DNA-binding transcription factor activity (GO:0051090), and cellular response to biotic stimulus (GO:0071216) (**Figure 6A**). The KEGG pathway related to CB can roughly be divided into modules of inflammation, immune responses, and cancer. The 20 core targets were closely related to signaling pathways, such as IL-17 signaling pathway (hsa04657), AGE-RAGE signaling pathway in diabetic complications (hsa04933), TNF signaling pathway (hsa04668), the NOD-like receptor signaling pathway (hsa04621), and the Toll-like receptor signaling pathway (hsa04620) (**Figure 6B**). The first 20 representative signaling pathways are shown in **Table 3**, and these pathways may be key pathways for treating CB. The data suggested that PG treatment of CB is a multichannel action. The 20 core targets were related to diseases, such as Pertussis, Tuberculosis, Salmonella infection, and Influenza A (**Figure 6B**). The top 10 pathways were searched in the KEGG database (https://www.kegg.jp/), and annotated with the PathwayBuilderTool_2.0 to obtain potential pathways for PG treatment of CB (**Figure 6C**). This analysis provided a novel approach for the limited treatment of CB.

**Table 3:** Top 20 clusters with their representative enriched terms.

Category	Description	Count	LogP	Gene
hsa04657	IL-17 signaling pathway	13	−26.89	CASP8, MAPK14, HSP90AA1, IL1B, IL4, IL6, JUN, MMP9, MAPK1, MAPK3, MAPK8, PTGS2, TNF
hsa04933	AGE-RAGE signaling pathway in diabetic complications	11	−21.30	CCND1, MAPK14, FN1, IL1B, IL6, JUN, MMP2, MAPK1, MAPK3, MAPK8, TNF
hsa04668	TNF signaling pathway	11	−20.86	CASP8, MAPK14, IL1B, IL6, JUN, MMP9, MAPK1, MAPK3, MAPK8, PTGS2, TNF
hsa04621	NOD-like receptor signaling pathway	11	−18.62	BCL2L1, CASP8, MAPK14, HSP90AA1, IL1B, IL6, JUN, MAPK1, MAPK3, MAPK8, TNF
hsa04620	Toll-like receptor signaling pathway	9	−16.24	CASP8, MAPK14, IL1B, IL6, JUN, MAPK1, MAPK3, MAPK8, TNF
hsa04660	T cell receptor signaling pathway	8	−14.00	MAPK14, IL2, IL4, IL10, JUN, MAPK1, MAPK3, TNF
hsa04068	FoxO signaling pathway	7	−11.04	CCND1, MAPK14, IL6, IL10, MAPK1, MAPK3, MAPK8
hsa04664	Fc epsilon RI signaling pathway	6	−10.83	MAPK14, IL4, MAPK1, MAPK3, MAPK8, TNF
hsa04912	GnRH signaling pathway	6	−10.02	MAPK14, JUN, MMP2, MAPK1, MAPK3, MAPK8
hsa04915	Estrogen signaling pathway	6	−9.85	HSP90AA1, JUN, MMP2, MMP9, MAPK1, MAPK3
hsa04010	MAPK signaling pathway	7	−9.03	MAPK14, IL1B, JUN, MAPK1, MAPK3, MAPK8, TNF
hsa04917	Prolactin signaling pathway	5	−8.58	CCND1, MAPK14, MAPK1, MAPK3, MAPK8
hsa04012	ErbB signaling pathway	5	−8.12	ERBB2, JUN, MAPK1, MAPK3, MAPK8
hsa04071	Sphingolipid signaling pathway	5	−7.43	MAPK14, MAPK1, MAPK3, MAPK8, TNF
hsa04722	Neurotrophin signaling pathway	5	−7.41	MAPK14, JUN, MAPK1, MAPK3, MAPK8
hsa04921	Oxytocin signaling pathway	5	−6.88	CCND1, JUN, MAPK1, MAPK3, PTGS2
hsa04370	VEGF signaling pathway	4	−6.82	MAPK14, MAPK1, MAPK3, PTGS2
hsa04066	HIF-1 signaling pathway	4	−5.88	ERBB2, IL6, MAPK1, MAPK3
hsa04024	cAMP signaling pathway	4	−4.72	JUN, MAPK1, MAPK3, MAPK8
hsa04662	B cell receptor signaling pathway	3	−4.57	JUN, MAPK1, MAPK3

### Computational Validation of Ingredient-Targets Interactions

**Figure 7:**
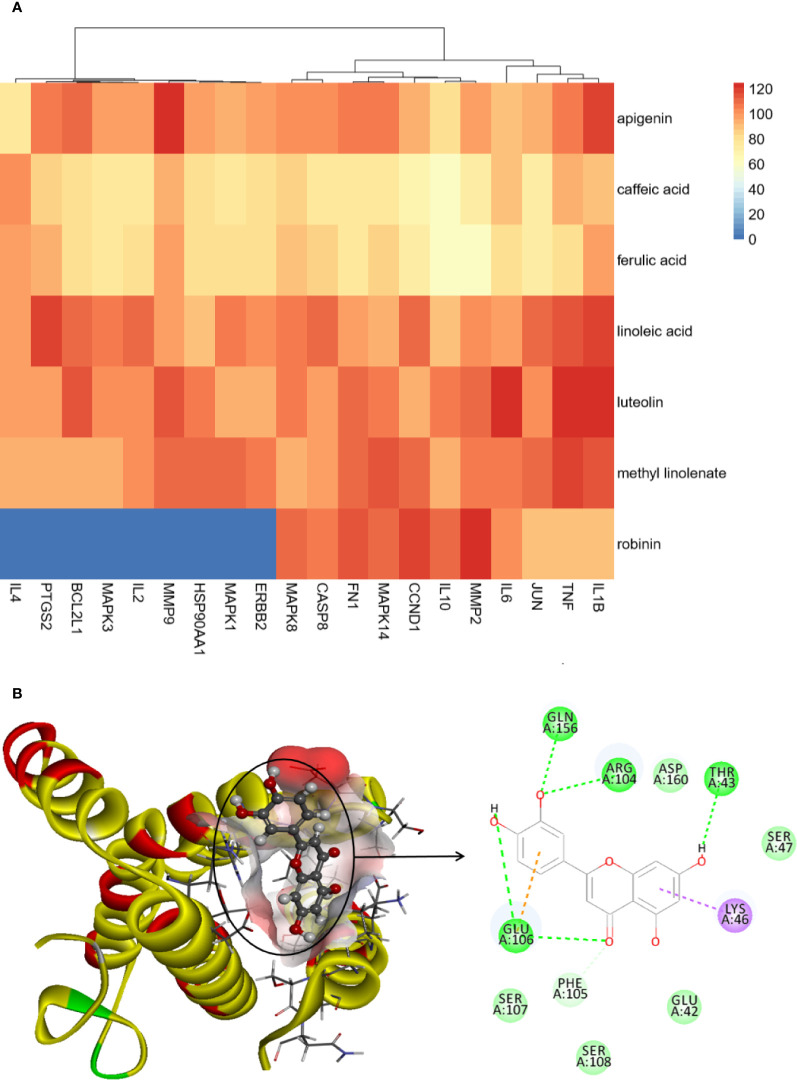
**(A)** Ingredient-core target docking scores. **(B)** Luteolin-IL-6.

Results of the docking score are presented in **Figure 7A** and **Schedule 6**. In general, the higher the molecular docking score, the stronger the binding ability of the receptor to ligand. The docking results showed that most core ingredients and core targets can be well combined. Luteolin had good affinity with IL-6, TNF, and IL-1β, which was consistent with the results of cell verification experiments, and showed that luteolin has good antiinflammatory effects. Furthermore, clustering results showed that IL-6, TNF, IL-1β, and Jun were classified. An example of molecular docking is presented in **Figure 7B** (Luteolin-IL-6). Luteolin can form hydrogen bonds with IL-6 at LYS A:171, HIS A:164, and GLU A:51, and hydrogen bonding is the main force that caused it to bind the active site.

Based on these data, we considered that the interaction between these core targets and core ingredients was the basis of biologic activity. This also proved that PG exerted its drug effect through the combined action of multitargets.

### Experimental Verification *In Vitro*

#### CCK-8 Assay

**Figure 8:**
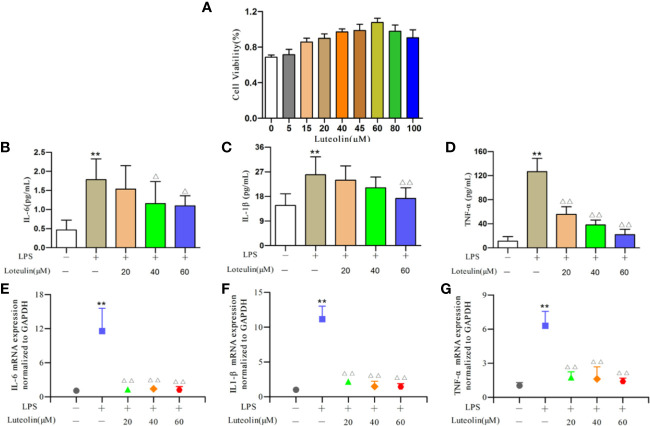
Effect of luteolin on RAW264.7 cells. RAW264.7 cells were incubated with lipopolysaccharide (LPS) (1 μg/ml) for 24 h and then treated with luteolin (20, 40, or 60 μM) for 24 h. The effects of luteolin **(A)** on the viability of RAW264.7 cells using the CCK-8 assay. Production of interleukin-6 (IL-6) **(B)**, tumor necrosis factor-α (TNF-α) **(C)**, and IL-1β **(D)** was determined by enzyme-linked immunosorbent assay (ELISA). Protein expression of IL-6 **(E)**, TNF-α **(F)**, and IL-1β **(G)** was determined by real-time quantitative polymerase chain reaction (qRT-PCR). **p < 0.01 versus blank control group. ^ΔΔ^p < 0.01 versus LPS-treated group. ^Δ^p < 0.05 versus LPS-treated group.

First, we determined the effects of different doses of luteolin on the viability of RAW264.7 cells using the CCK-8 assay (**Figure 8A**). Luteolin at ≥20 μM or ≤80 μM had high cell viability (>85%). Therefore, for subsequent experiments, three concentrations were selected (20, 40, 60 μM).

#### Validation of Targets

Luteolin is a flavonoid ingredient with a high content in PG (Sung et al., 2012; Piao et al., 2017), and has antiinflammatory, antitumor, antioxidant activity, and other pharmacological effects (Wang et al., 2013; Aziz et al., 2018; Xu et al., 2019). To further evaluate the results obtained by systematic pharmacologic analyses, luteolin was selected from PG to examine potential antiinflammatory effects using LPS (1 μg/ml)-stimulated RAW264.7 cells. We employed ELISA and qRT-PCR for IL-6, TNF-α, and IL-1β to confirm the predicted antiinflammatory effects of luteolin.

The contents of IL-6, TNF-α, and IL-1β in RAW264.7 cells increased significantly (all *p*<0.01, **Figure 8**) after LPS induction. With different concentrations of luteolin treatment, the content of each index decreased to varying degrees when compared with the model group. Regarding the IL-6 content, the medium- and high-dose groups showed a significant downward trend (*p*<0.05, **Figure 8B**). For IL-1β, only the high-dose group showed significant differences (*p*<0.01, **Figure 8C**). In addition, the TNF-α content of each dose group showed extremely significant difference (all *p*<0.01) (**Figure 8D**).

The results of qRT-PCR showed that the expression of IL-6, TNF-α, and IL-1β mRNA in the LPS-stimulated group cells was significantly higher compared to the blank group (all *p*<0.01, **Figure 8**). The expression of these genes decreased significantly (all *p*<0.01, **Figure 8**) after luteolin treatment.

In conclusion, these data indicated that luteolin from PG may inhibit the inflammatory response by regulating the expression of IL-6, IL-1β, and TNF-α thereby treating CB. *In vitro* studies provided additional information for screening ingredients with potential antiinflammatory effects, and demonstrated the rationality of molecular docking results and the reliability of screening strategies based on systematic pharmacology.

## Discussion

In clinical practice, PG is one of the most commonly used Chinese medicines and has been used in China for thousands of years. PG has a significant therapeutic effect on respiratory diseases, and is commonly used to treat CB (Sun et al., 2010; He et al., 2013), pneumonia (Zhang et al., 2013; Sui et al., 2015), and lung cancer (Huang M. Y. et al., 2019; Li Y. et al., 2019). CB is a common clinical disease, and in medical practice, antibacterial drugs, antiallergic drugs, and other Western medicines are often used. Because CB has a long course of disease, bacterial resistance is often observed, leading to a condition that is not effectively controllable by many drugs (Jin, 2019). In clinical applications, TCM can achieve similar or even better therapeutic effects than Western medicine. In previous studies, it has been shown that TCM, including PG, have biological effects on CB (Chen et al., 2010; Chen et al., 2013; He et al., 2013), however, due to multiingredient and multitarget characteristics of Chinese medicine, the specific underlying mechanism of the pharmacodynamic effects is still unclear. Therefore, the application of network pharmacology methods, combined with active ingredient screening, drug targets, and network and pathway analysis to investigate the underlying mechanism of action of PG in the treatment of CB, is imperative.

In this study, 36 nonvolatile ingredients were identified from PG using UPLC-Q-TOF-MS/MS technology. Based on the basic properties of the ingredients and combined with literature studies, 10 major active ingredients, such as Platycodin D and luteolin were selected, they exert a pharmacological effect by affecting 144 overlapping genes that play a role in the treatment of CB. The PPI network showed that IL-6 and TNF were the most relevant targets for PG in the treatment of CB. The D-I-G-D network showed that the same target may interact with multiple ingredients. For example, TNF can bind to luteolin, apigenin, and robinin, whereas IL-6 can bind to linoleic acid and luteolin. This shows that multiple active ingredients may act on the same target. In addition, we found that luteolin was related to IL-6, TNF, IL-1β, MMP9, JUN, CCND1, ERBB2, IL-4, IL-2, and MMP2. Furthermore, platycodin D was related to CCNE1 and HMOX1, thereby indicating that PG can act on multiple targets through the same active ingredient. Thus, these findings proved that PG had a multiingredient and multitarget synergy effect and exerted its efficacy by providing a basis for studying multiingredient multitarget synergy.

Enrichment analysis of GO and KEGG pathways on 20 core targets was performed, and we obtained 710 GO biological processes and 113 KEGG pathways. Among them, GO function enrichment results were mostly related to cell inflammation, oxidative stress response, proliferation and apoptosis, and energy metabolism. We speculated that the response to LPS may be the most important biological process of PG in the treatment of CB. KEGG pathway enrichment results showed that the top 5 signaling pathways included the IL-17 signaling pathway, AGE-RAGE signaling pathway in diabetic complications, TNF signaling pathway, NOD-like receptor signaling pathway, and Toll-like receptor signaling pathway, which were highly involved the imbalanced “inflammation-immune” system of CB. Especially, three major hubs, including IL-6, IL-1β, and TNF were involved in the IL-17 signaling pathway and TNF signaling pathway, and directly interacted with each other. In addition, expectoration, cough, and wheezing are the main symptoms of CB (Zhang, 2019). Among the drug-disease core targets, IL-6, IL-1β, and TNF-α have been identified as the main therapeutic targets for expectoration, cough, and wheezing (Liu and Chen, 2017; Huang H. F. et al., 2019). Therefore, for further experimental verification, IL-6, IL-1β, and TNF-α were selected as candidate targets of PG against CB. Finally, the results of molecular docking and *in vitro* verification experiments proved that PG can effectively treat CB by inhibiting inflammatory responses.

## Conclusion

In the current study, we combined chemical ingredient analysis, target prediction, network calculation, and experimental validations to identify the chemical constituents contained in PG, and offered the convincing evidence that PG may act against CB by reducing inflammation. These findings provided the experimental basis for the scientific connotation and the clinical application of PG against CB.

## Data Availability Statement

The raw data supporting the conclusions of this article will be made available by the authors, without undue reservation.

## Ethics Statement

The authors declare that the procedures followed were in accordance with the regulations of the relevant clinical research ethics committee and with those of the Code of Ethics of the World Medical Association (Declaration of Helsinki).

## Author Contributions

YD integrated the data and wrote the manuscript. HR completed the ingredient identification. XWY and LX accomplished the pharmacological study. ML and YL executed the literature search. SY directed the data processing. MY and XDY implemented corrections in the manuscript. JZ conceptualized and designed the experimental plan.

## Funding

This study was supported by the National Key R&D Program - Special Topics for Modernization of Traditional Chinese Medicine (No. 2018YFC1707206, No. 2018YFC1707200), Key R&D Program of Jiangxi Province (No. 20192BBG70073), National Natural Science foundation of China (No. 81560651), Jiangxi Province’s double first-class discipline (Traditional Chinese Medicine) construction project (No. JXSYLXK-ZHYAO039/141), and Doctoral Research start-up Fund (No. 2018WBZR009).

## Conflict of Interest

The authors declare that the research was conducted in the absence of any commercial or financial relationships that could be construed as a potential conflict of interest.
