# Supplementary material for: Integrated Phytochemical Analysis Based on UPLC-Q-TOF-MS/MS, Network Pharmacology, and Experiment Verification to Explore the Potential Mechanism of Platycodon grandiflorum for Chronic Bronchitis
Source: Front Pharmacol. 2020 Sep 8;11:564131. doi: 10.3389/fphar.2020.564131 (PMC7506058; doi:10.3389/fphar.2020.564131)
Supplement: Supplementary file 2 [file DataSheet_2.docx]

Research highlights

- 36 compounds identified from Platycodon grandiflorum.
- Prediction of active ingredients, core targets, and potential signaling pathways of Platycodon grandiflorum in the treatment of chronic bronchitis.
- The reliability of the core targets was evaluated through *in vitro* studies.
